# Supplementary material for: Childhood parasitic infections and gastrointestinal illness in indigenous communities at Lake Atitlán, Guatemala
Source: PeerJ. 2021 Nov 17;9:e12331. doi: 10.7717/peerj.12331 (PMC8605761; doi:10.7717/peerj.12331)
Supplement: Supplemental Information 1 — Participants received educational training in how to reduce risks for their children and others in their family from water borne diseases, including information on water treatment options. [file peerj-09-12331-s001.pdf]

Buenas prácticas de higiene

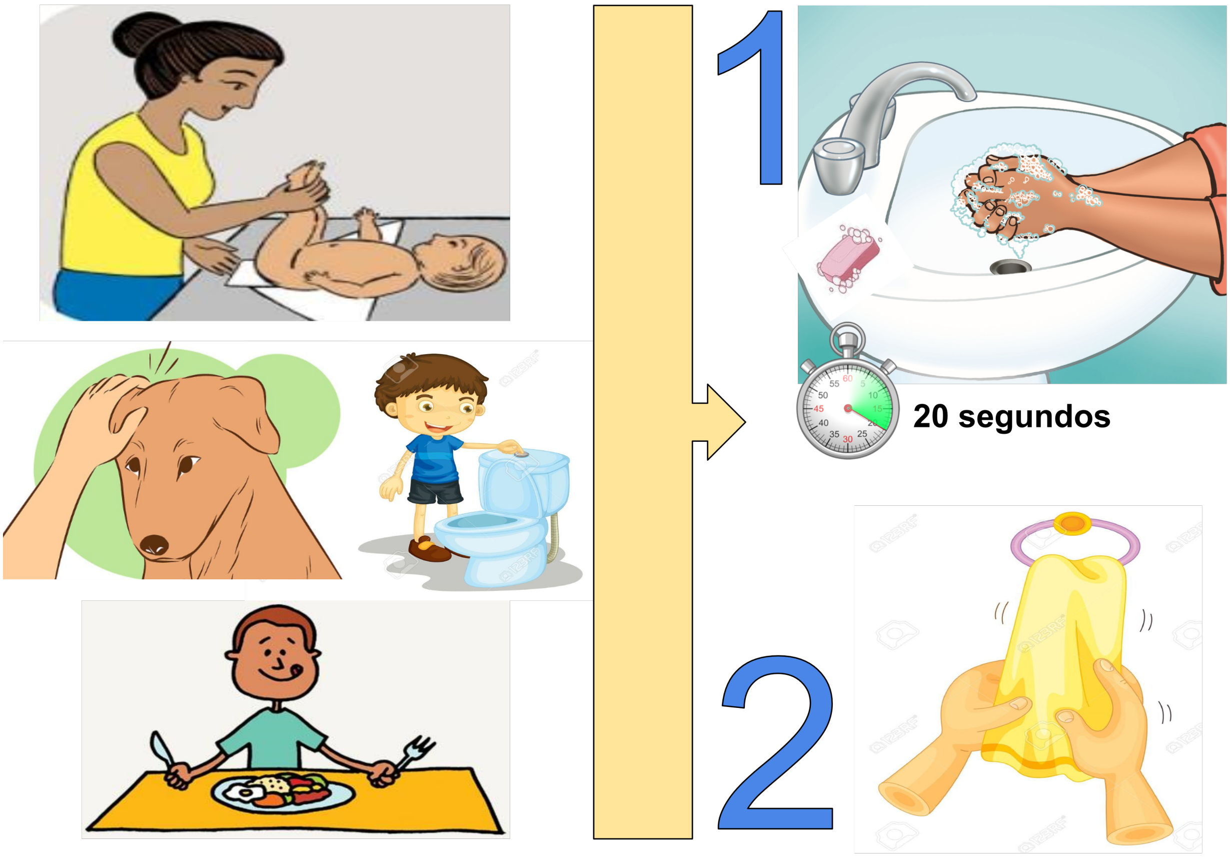

Purificación de agua en hogar

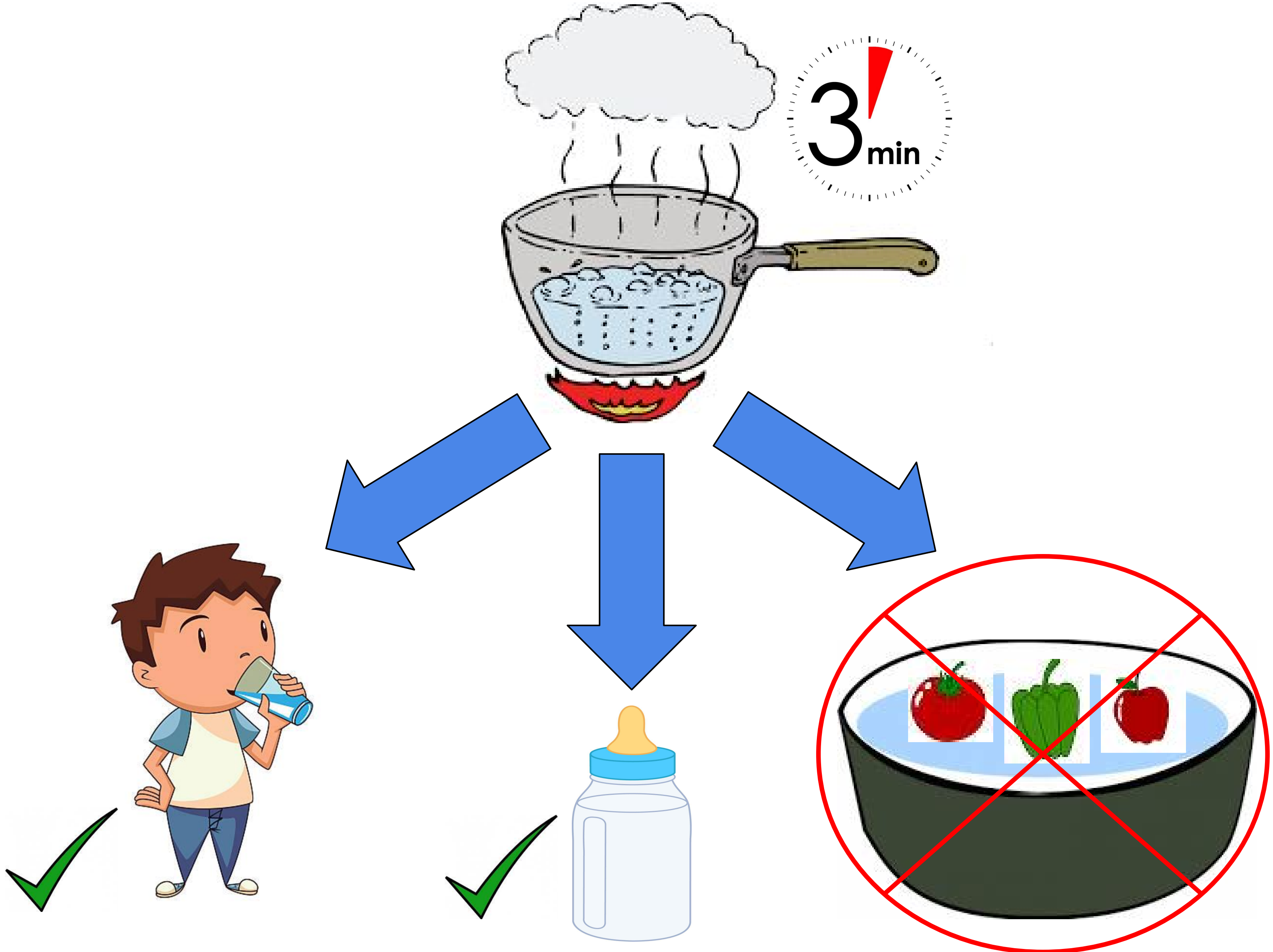

Preparación de comida

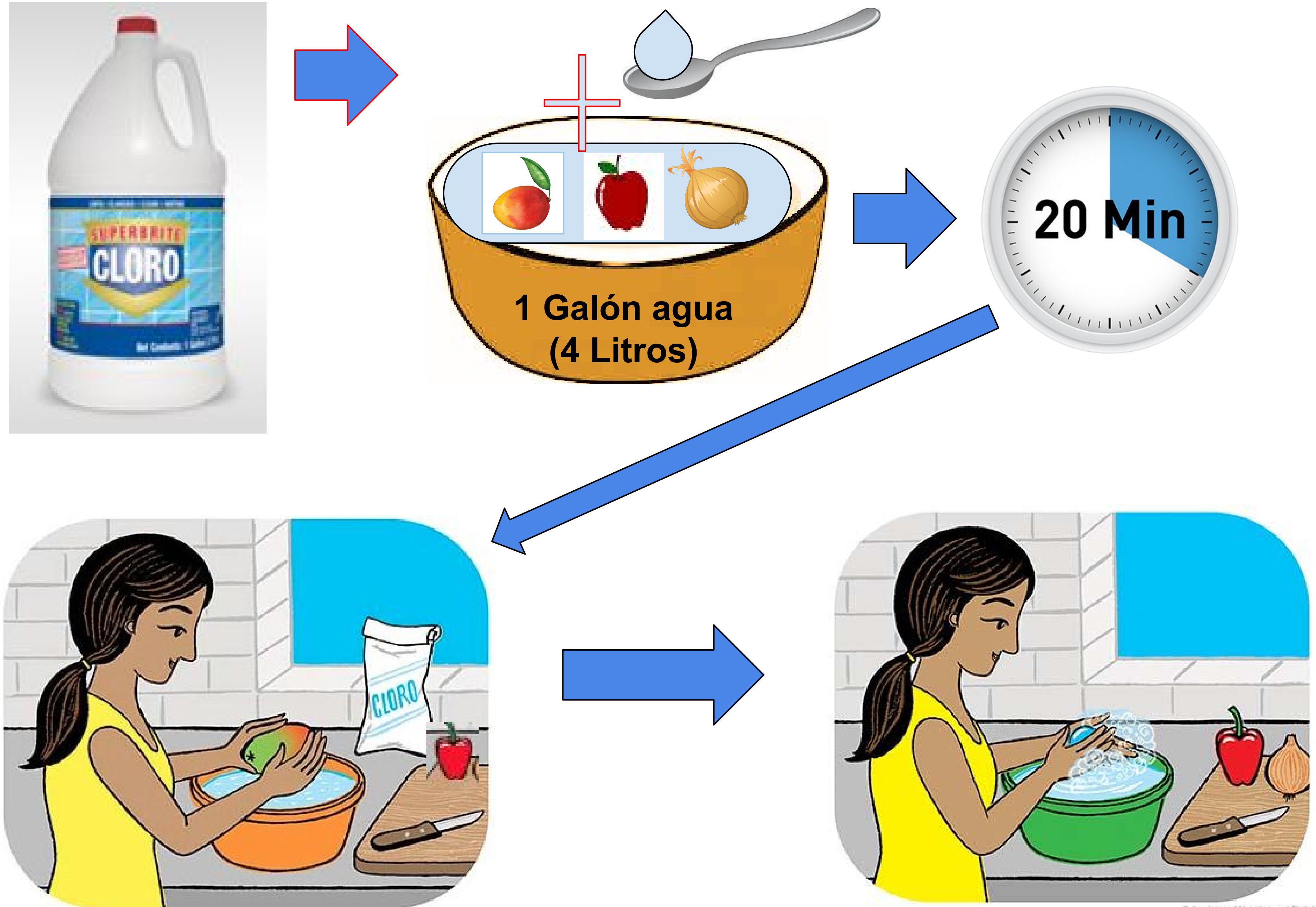

© Academy of Nutrition and Dietetics Foundation

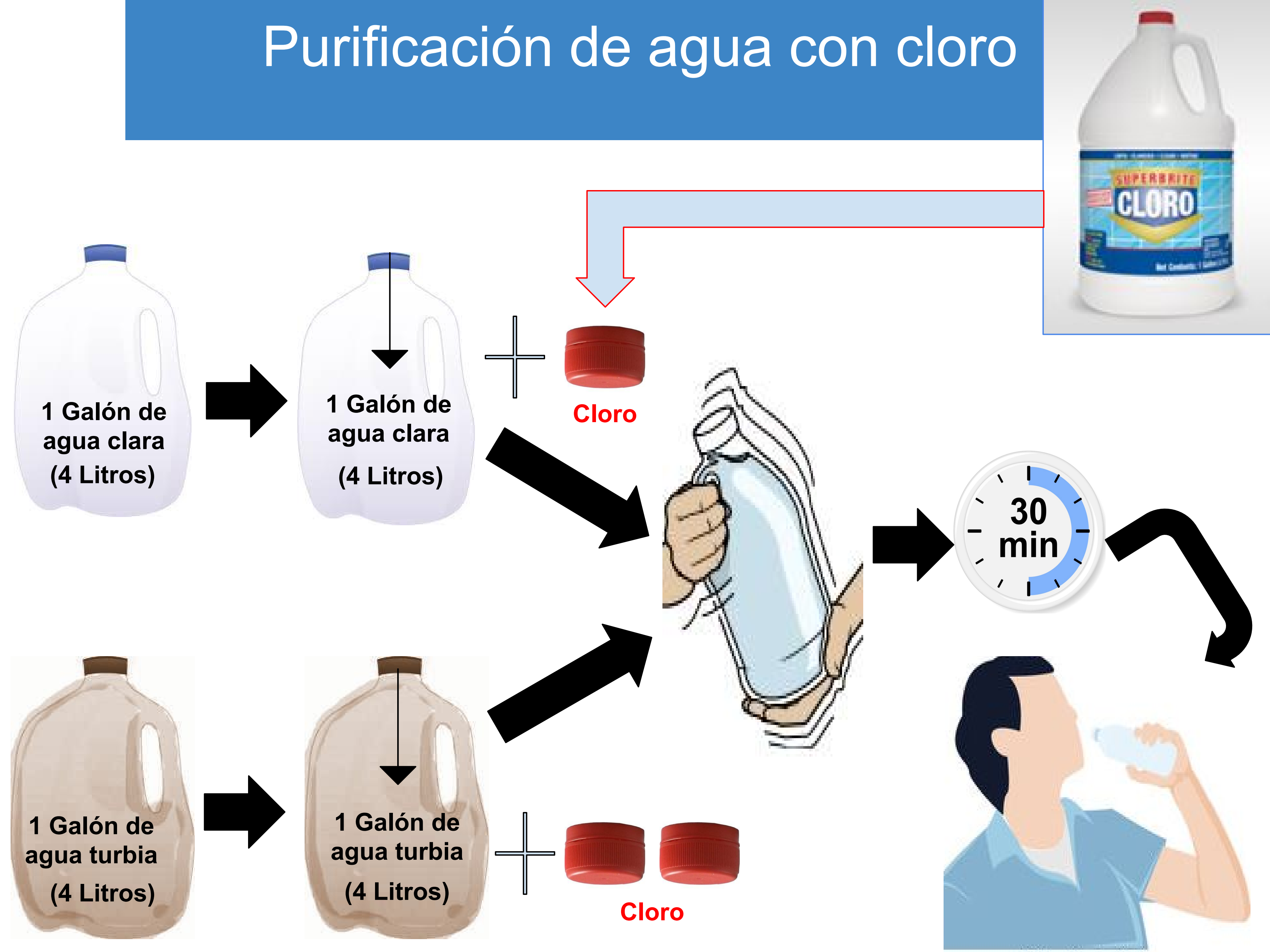
Purificación de agua con cloro
SUPERBRITE
CLORO
1 Galón de agua clara (4 Litros)
1 Galón de agua clara (4 Litros)
Cloro
30 min
1 Galón de agua turbia (4 Litros)
1 Galón de agua turbia (4 Litros)
Cloro
