# Supplementary material for: Childhood parasitic infections and gastrointestinal illness in indigenous communities at Lake Atitlán, Guatemala
Source: PeerJ. 2021 Nov 17;9:e12331. doi: 10.7717/peerj.12331 (PMC8605761; doi:10.7717/peerj.12331)
Supplement: Supplemental Information 2 — The attending physician at the participating clinic was in charge of providing and monitoring treatment, with medication provided as a donation by the project courtesy of the Rotary E Club of Atitlán, Panajachel, Guatemala. [file peerj-09-12331-s002.docx]

**Appendix B. Medication Protocol:**

The attending physician at the participating clinic was in charge of providing and monitoring treatment, with medication provided as a donation by the project courtesy of the Rotary E Club of Atitlán, Panajachel, Guatemala.

- Patients testing positive for ***Giardia* or *Entamoeba*** will receive metronizadole, 35-50 mg/kg/day by mouth to be taken 3 times per day, for 3-10 days. Patients with ***Entamoeba*** in the luminal stage will receive diloxanide furoate, 25 mg/kg to be taken by mouth three times per day, for 3-10 days.
- Patients testing positive for ***Cryptosporidium*** will receive nitazoxanide 100 mg (children aged <3 years) or 200 mg (children aged 4-5 yrs), to be taken twice a day for three days.

No major risks were associated with such treatment, but potential risks discussed included: 1) allergic reaction (rashes or breathing problems which require immediate cessation of use of antibiotics) 2) increased bowel pain for treatment period but should be distinguished from prolonged increase or additional signs (such as bloody diarrhea or vomiting). An accurate weight was obtained to assure that over or under dosing would not occur.
