# Supplementary material for: Childhood parasitic infections and gastrointestinal illness in indigenous communities at Lake Atitlán, Guatemala
Source: PeerJ. 2021 Nov 17;9:e12331. doi: 10.7717/peerj.12331 (PMC8605761; doi:10.7717/peerj.12331)
Supplement: Supplemental Information 3 — Questionnaire utilized in study under IRB approval. [file peerj-09-12331-s003.docx]

**Appendix C: WASH Questionnaire**

**Breve Cuestionario sobre Salud, Agua, Saneamiento e Higiene**

*Instrucciones para el profesional de salud y representante del estudio: Por favor asegúrese que el consentimiento apropiado se haya obtenido antes de documentar cualquier información. Si el consentimiento se retira en cualquier momento, por favor destruir esta hoja y pedirle al representante del estudio que se retire de la sala de examinar a los pacientes para continuar con la cita. Después de completar la hoja del examen físico y el cuestionario, el representante del estudio debería salir de la sala de examinar a los pacientes.*

ID del niño: ___________________ *(Esto será completado por los investigadores antes de ser distribuidos en las clínicas)*

Edad del niño: ___________________

Sexo: F M

1. ¿Su hijo(a) sufre de alguna condición gastrointestinal crónica? (Estos incluyen síndrome de colon irritable, enfermedad de Crohn, alergias alimenticias, o enfermedad celiaca):
   1. Si
   2. No
2. En casa, ¿de dónde obtienen el agua para tomar? (Circule todas las que aplican.
   1. Agua del lago
   2. Agua de manantial o nacimiento
   3. Agua municipal
   4. Agua embotellada
3. ¿Trata de alguna forma el agua que usan en su casa? (Si responde que no, pasar a pregunta 5).
   1. Si (pase a pregunta 4)
   2. No (pase a pregunta 5)
4. ¿De qué forma trata el agua que usan en su casa?
   1. Hervirla
   2. Filtro de tela
   3. Clorarla
   4. Filtro de arcilla
   5. Filtro de carbón
   6. Otro (Especificar): ____________________________
5. ¿Usa esta agua para lavar frutas y verduras?
   1. Si
   2. No
   3. A veces
   4. Si usa agua obtenida de otra fuente para lavar frutas y verduras, especifique: _______________
6. ¿Usa esta agua para lavar los platos?
   1. Si
   2. No
   3. A veces
   4. Si usa agua obtenida de otra fuente para lavar los platos, especifique:_____________________
7. ¿Tiene animales en su casa o patio? Por favor enumere los animales que tiene: _____________________________________________________________________________________
8. ¿Qué tan seguido a lo largo de un año tiene diarrea su hijo(a) (que está participando)?
   1. Nunca
   2. Varias veces en un año
   3. Una vez al mes
   4. Varias veces al mes
   5. Una vez a la semana
   6. Varias veces en la semana
   7. No sabe
9. ¿Qué tan seguido a lo largo de un año vomita su hijo(a) (que está participando)?
   1. Nunca
   2. Varias veces en un año
   3. Una vez al mes
   4. Varias veces al mes
   5. Una vez a la semana
   6. Varias veces a la semana
   7. No sabe
10. ¿Qué tan seguido a lo largo de un año le da fiebre a su hijo(a) (que está participando)?
    1. Nunca
    2. Varias veces en un año
    3. Una vez al mes
    4. Varias veces al mes
    5. Una vez a la semana
    6. Varias veces a la semana
    7. No sabe
11. ¿Tiene otros niños en su casa? (Si la respuesta es sí, anote el número) (*Niños son menores de 18 años)*
    1. Si: Anote el número: ________
    2. No (pase a pregunta 12)
12. Si tiene otros niños en su casa, ¿a ellos les da diarrea frecuentemente?
    1. Si
    2. No
13. ¿Qué tan seguido les da diarrea a los otros niños que viven en su casa? (Marque para cada niño)
    1. Nunca
    2. Varias veces en un año
    3. Una vez al mes
    4. Varias veces al mes
    5. Una vez a la semana
    6. Varias veces en la semana
    7. No sabe
14. ¿Los demás niños en su casa vomitan con frecuencia?
    1. Si
    2. No
15. ¿Qué tan seguido les dan vómitos a los otros niños que viven en su casa? (Marque para cada niño)
    1. Nunca
    2. Varias veces en un año
    3. Una vez al mes
    4. Varias veces al mes
    5. Una vez a la semana
    6. Varias veces en la semana
    7. No sabe
16. ¿Qué tipo de facilidades sanitarias (inodoro/letrina si hay) usan con más frecuencia los miembros de su familia? (MARQUE UNO, use el panfleto de guía para enseñarle al padre/madre que responde)
    1. Vaciar o lavar/echar agua al inodoro o letrina
    2. Letrina de fosa mejorada y ventilada (VIP)
    3. Letrina de pozo con loza
    4. Letrina de pozo sin loza/abierta
    5. Compostaje / retrete seco o letrina
    6. Inodoro suspendido / letrina colgante
    7. Servicio o letrina de cubo (donde las heces se eliminan manualmente)
    8. No tienen facilidades / defecan en el campo o en bolsas plásticas
    9. Otra
    10. No sabe/No responde
17. ¿Este inodoro/letrina se comparte con otras familias?
    1. Si (pasar a pregunta 18)
    2. No (pasar a pregunta 19)
    3. No sabe
18. ¿Cuántas otras casas usan este inodoro/letrina? Anote el número: _________
19. ¿Puede cualquier persona usar este inodoro/letrina?
    1. Si
    2. No
    3. No sabe
20. La última vez que [NOMBRE DEL NIÑO MAS PEQUEÑO] defecó, ¿cómo desecharon las heces? (MARQUE UNO)
    1. El niño uso inodoro/letrina
    2. Se desaguaron en el inodoro o letrina
    3. Se echaron en el desagüe o zanja
    4. Se tiraron a la basura
    5. Se enterraron
    6. Se dejaron en el patio al aire libre
    7. Se dejaron afuera de la casa
    8. Otro
    9. No sabe/No responde
