# Supplementary material for: Childhood parasitic infections and gastrointestinal illness in indigenous communities at Lake Atitlán, Guatemala
Source: PeerJ. 2021 Nov 17;9:e12331. doi: 10.7717/peerj.12331 (PMC8605761; doi:10.7717/peerj.12331)
Supplement: Supplemental Information 4 [file peerj-09-12331-s004.docx]

**Appendix D: Physical Exam Guide**

**Guía de Examen Físico**

*Instrucciones para el profesional de salud y representante del estudio: Por favor asegúrese que el consentimiento apropiado se haya obtenido antes de documentar cualquier información. Si el participante cambia de parecer durante los procedimientos del estudio, suspender las actividades del mismo y colocar una nota en el consentimiento informado. Después de completar la hoja del examen físico y el cuestionario, el representante del estudio debería salir de la sala de examen a los pacientes.*

ID del niño: ___________________ *(Esto será completado por los investigadores antes de ser distribuidos en las clínicas)*

1. Edad del niño: ___________________
2. Sexo: F M
3. Número de niños en la familia: ____________________
4. Seleccione la fuente de agua para tomar que normalmente usa (Marque todas las que apliquen):
5. Agua del lago Atitlán
6. Agua de manantial o nacimiento
7. Agua municipal, chorro privado
8. Agua municipal, chorro público
9. Agua en botella, garrafón o bolsita plástica
10. Notas adicionales: __________________________________________________________________________________________________________________________________________________
11. Seleccione cuál de estos problemas o síntomas que padece su niño (si los tiene), le preocupan. Marque todas las que apliquen.
    1. Diarrea
    2. Vómitos
    3. Nausea
    4. Dolor abdominal
    5. Fiebre
    6. Escalofríos
    7. Ninguna de las anteriores
    8. Notas adicionales:

________________________________________________________________________________________________________________________________________________

1. ¿Se recolectó la muestra de heces durante el examen?
   1. Si
   2. No
2. Anote la talla del niño(a) (en cm): ___________
3. Anote el peso del niño(a) (en kg): ____________
4. Anote la temperatura del niño(a) (en °C): ________
5. Anote la frecuencia cardiaca del niño(a) (en latidos por minuto): __________
6. Anote las respiraciones del niño(a) (en respiraciones por minuto): _________
7. ¿Tiene sonidos respiratorios (incluye esfuerzo de respiración, sonidos estertores o estridores, o sibilancias)? S/N Favor de describir tipo si hay: ___________________________________
8. ¿Tiene el niño síntomas de deshidratación? S/N. Subraye los síntomas que aplican: ---

- tiempo de relleno capilar > 2s
- ojos hundidos
- membranas mucosas pálidas
- taquicardia
- pulsos anormales
- respiraciones anormales
- disminución de la elasticidad de piel
  1. Si, anote el grado :
     1. Leve (3 a 6%) (uno a dos de los síntomas)
     2. Moderada (>6%) (tres a seis síntomas)
  2. No tiene síntomas de deshidratación

1. Por favor anote cualquier información adicional sobre la salud general del niño(a):
