# Supplementary material for: Childhood parasitic infections and gastrointestinal illness in indigenous communities at Lake Atitlán, Guatemala
Source: PeerJ. 2021 Nov 17;9:e12331. doi: 10.7717/peerj.12331 (PMC8605761; doi:10.7717/peerj.12331)
Supplement: Supplemental Information 6 [file peerj-09-12331-s006.docx]

**Table S1. Physical exam data descriptive statistics for males and females**. **A significant difference (student t-test) was observed between males and females for body mass index, but not for other values.

| **Descriptor** | ***p-value (t-test)*** | **Sex** | **n** | **Mean** | **Standard Deviation** | **Median** |
| --- | --- | --- | --- | --- | --- | --- |
| **Age (Months)** | *0.38* | F | 186 | 35 | 16.5 | 36 |
|  |  | M | 215 | 33.5 | 17.4 | 36 |
| **Height (cm)** | *0.21* | F | 183 | 88.5 | 15.4 | 90 |
|  |  | M | 214 | 86.6 | 15.1 | 89.8 |
| **Weight (kgs)** | *0.77* | F | 184 | 12.5 | 3.3 | 12.7 |
|  |  | M | 214 | 12.6 | 3.4 | 13 |
| **BMI**** | *0.01*** | F | 183 | 16.2 | 3.3 | 16 |
|  |  | M | 214 | 17 | 3.2 | 16.5 |
| **Temperature (deg C)** | *0.76* | F | 174 | 36.7 | 0.6 | 36.7 |
|  |  | M | 194 | 36.7 | 0.6 | 36.8 |
| **Heart rate (bpm)** | *0.7* | F | 169 | 86.6 | 24.8 | 80 |
|  |  | M | 179 | 87.5 | 19.6 | 84 |
| **Respiratory Rate (bpm)** | *0.77* | F | 169 | 22.9 | 11 | 20 |
|  |  | M | 182 | 23.3 | 12.2 | 20 |

**Table S2. Physical exam data descriptive statistics across communities.** *A significant difference (ANOVA) was observed across communities for BMI, height, and weight, but likely is related to significant differences in ages recruited across communities.

| **Descriptor** | **P-value for F statistic** | **Locations** | **n** | **Mean** | **Standard Deviation** | **Median** |
| --- | --- | --- | --- | --- | --- | --- |
| **Age (months)** | *0.002** | AE | 49 | 29.8 | 16.1 | 24 |
|  |  | PA | 30 | 42.2 | 12.8 | 48 |
|  |  | SA | 34 | 32.9 | 15.3 | 36 |
|  |  | SJ | 75 | 38.8 | 15.2 | 48 |
|  |  | SL | 123 | 33.5 | 18 | 36 |
|  |  | SP | 60 | 29.7 | 18.5 | 29 |
|  |  | TL | 30 | 35.1 | 16.1 | 42 |
| **Height (cm)** | *0.0000687** | AE | 49 | 84.5 | 14.6 | 86.4 |
|  |  | PA | 30 | 94.9 | 13.9 | 96 |
|  |  | SA | 32 | 89.5 | 13.2 | 90 |
|  |  | SJ | 75 | 89.8 | 13.9 | 92.7 |
|  |  | SL | 123 | 85.5 | 13.1 | 86 |
|  |  | SP | 58 | 82.2 | 13.1 | 80.8 |
|  |  | TL | 30 | 95.5 | 26 | 91.4 |
| **Weight (kgs)** | *0.0198** | AE | 49 | 11.7 | 3.8 | 11.8 |
|  |  | PA | 30 | 14.1 | 2.8 | 14.2 |
|  |  | SA | 33 | 12.5 | 3.5 | 12.8 |
|  |  | SJ | 75 | 13.2 | 2.9 | 13.5 |
|  |  | SL | 123 | 12.4 | 3.5 | 12.4 |
|  |  | SP | 58 | 12 | 3.6 | 11.4 |
|  |  | TL | 30 | 13 | 2.9 | 14.1 |
| **BMI** | *0.0123** | AE | 49 | 16 | 2.2 | 16 |
|  |  | PA | 30 | 16 | 3.5 | 15.5 |
|  |  | SA | 32 | 15.4 | 2.7 | 15.3 |
|  |  | SJ | 75 | 16.6 | 3.1 | 16.3 |
|  |  | SL | 123 | 16.9 | 2.8 | 16.3 |
|  |  | SP | 58 | 17.7 | 3.4 | 17.4 |
|  |  | TL | 30 | 16 | 5.6 | 16.9 |

**Fig S1. Box and whiskers notched plots of ages of both male and female study participants at each study location.** Black plots depict female distribution and grey depict male distribution.


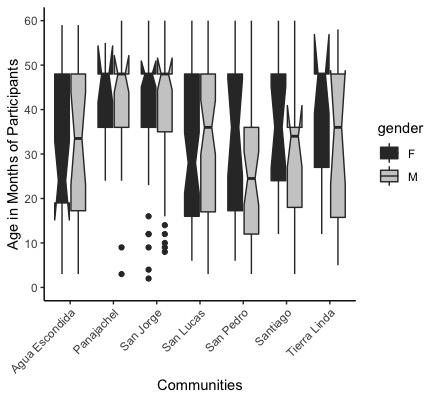


**Table S3. Single sample fecal coliforms and *Escherichia coli* counts at selected sites of drinking water intake at each municipality and at a tap water source.** 50 mL samples were kept on ice in a cooler and were immediately plated on return to the laboratory, utilizing 3M™ Petrifilm™ Rapid *E. coli*/Coliform Count Plates following AOAC guidelines (Bird et al., 2020). Samples were incubated for 24 hours +/- 2 hours at 35 degrees Celsius +/- 1 degree. Blue colonies with gas bubbles were counted as E coli, and red colonies with gas bubbles were counted as fecal coliforms.

| **Site** | **Source of Water for Site** | **Description** | **Date** | **Coliforms (counts per mL)** | ***E coli* (counts per mL)** |
| --- | --- | --- | --- | --- | --- |
| **San Lucas** | **lake water** | pipe for drinking water intake | 27-Sep-17 | 211 | 1 |
|  |  | household tap receiving “treated” water | 27-Sep-17 | 102 | 2 |
| **Santiago** | **lake water** | pipe for drinking water intake | 29-Sep-17 | 6 | 2 |
|  |  | household tap receiving "treated" water | 29-Sep-17 | 52 | 4 |
| **San Pedro** | **lake water** | pipe for drinking water intake | 29-Sep-17 | 330 | 170 |
|  |  | school tap receiving "treated” water | 29-Sep-17 | 33 | 0 |
| **Agua Escondida** | **spring water** | spring water source pre-treatment | 28-Sep-17 | 490 | 40 |
|  |  | post chlorination treatment by municipality | 28-Sep-17 | 0 | 0 |
| **San Jorge** | **spring water** | tap water for pre-school from spring source | 28-Sep-17 | 12 | 0 |
|  |  | post Sawyer filter at pre-school | 28-Sep-17 | 0 | 0 |
| **Tierra Linda** | **spring water** | tap water for pre-school from spring source | 28-Sep-17 | 0 | 0 |
|  |  | post Sawyer filter at pre-school | 28-Sep-17 | 49 | 0 |
| **Panajachel #1** | **spring water** | tap water for Panajachel | 28-Sep-17 | 2 | 0 |
|  |  | post-treatment (carbon filter) at pre-school | 28-Sep-17 | 7 | 0 |
| **Santa Catarina** | **lake water** | pre-treatment at lake water source | 30-Sep-17 | 66 | 13 |
|  |  | post-treatment (chlorination and UV) | 30-Sep-17 | 5 | 0 |
| **Panajachel #2** | **spring water** | pre-treatment tap water at local hotel on lake | 30-Sep-17 | 10 | 0 |
|  |  | post-treatment (clay pot filter) | 30-Sep-17 | 0 | 0 |
